# Unzippers, Resolvers and Sensors: A Structural and Functional Biochemistry Tale of RNA Helicases

**DOI:** 10.3390/ijms16022269

**Published:** 2015-01-22

**Authors:** Ana Lúcia Leitão, Marina C. Costa, Francisco J. Enguita

**Affiliations:** 1 Departamento de Ciências e Tecnologia da Biomassa, Faculdade de Ciências e Tecnologia, Universidade Nova de Lisboa, Quinta da Torre, Campus de Caparica, 2829-516 Caparica, Portugal; E-Mail: aldl@fct.unl.pt; 2 Instituto de Medicina Molecular, Faculdade de Medicina, Universidade de Lisboa, Av. Prof. Egas Moniz, 1649-028 Lisboa, Portugal; E-Mail: marinacosta@fm.ul.pt

**Keywords:** RNA helicase, structural biology, protein-RNA complex, DEAD-box protein, innate immunity, RIG-I

## Abstract

The centrality of RNA within the biological world is an irrefutable fact that currently attracts increasing attention from the scientific community. The panoply of functional RNAs requires the existence of specific biological caretakers, RNA helicases, devoted to maintain the proper folding of those molecules, resolving unstable structures. However, evolution has taken advantage of the specific position and characteristics of RNA helicases to develop new functions for these proteins, which are at the interface of the basic processes for transference of information from DNA to proteins. RNA helicases are involved in many biologically relevant processes, not only as RNA chaperones, but also as signal transducers, scaffolds of molecular complexes, and regulatory elements. Structural biology studies during the last decade, founded in X-ray crystallography, have characterized in detail several RNA-helicases. This comprehensive review summarizes the structural knowledge accumulated in the last two decades within this family of proteins, with special emphasis on the structure-function relationships of the most widely-studied families of RNA helicases: the DEAD-box, RIG-I-like and viral NS3 classes.

## 1. Introduction

RNA molecules often need to fold into specific three-dimensional structures in order to exert their biological functions. RNA folding is an intrinsically difficult problem which is commonly prevented by two major RNA features: the chemical characteristics of RNA make it “prone to misfold”, thereby becoming trapped into inactive energy minima; and the functional conformations are sometimes not energetically favored over the overall universe of folding intermediates [1]. For these reasons, cells require either the presence of controlled chemical environments or the assistance of specialized proteins to ensure the stabilization and proper RNA folding [2]. Moreover, within the cellular environment, many other external factors can be determinant for productive RNA folding. This process is also likely to be influenced by other mechanisms including transcription and translation [3].

X-ray crystallography studies of selected small and medium-size RNA molecules determined that the rules governing RNA folding are complex and comparable to those observed in proteins [4,5]. With the exception of the substitution of thymine by uracil, the same nucleotide DNA building blocks are also present in RNA, thus, a similar pattern of Watson-Crick base pairing is expected. However, the structure of RNA molecules is more diverse than the simple monotonous DNA helix, and it is often composed of short secondary structure elements packed into tertiary arrangements [6,7]. Moreover and unlike DNA, RNA double helices do not show structural polymorphisms, adopting only the A-form. This preferential conformation is induced by the presence of a 2'-hydroxyl group in the ribose ring, which alters the flexibility of the phosphodiester backbone favoring the A-form in duplex RNA but also facilitating sampling of an extended range of tertiary structures [8]. RNA structure is characterized by a panoply of secondary elements secured not only by Watson-Crick interactions but also by non-canonical base pairing events which are facilitated by the chemical versatility of the RNA chain and involved in high-degree intramolecular interactions [9]. Pseudo-knots, hairpin-loops, bulges, and kissing-hairpins, are some of the most common structural elements, which could be further packed into potentially more intricate tertiary structures that are frequently related to the RNA function [10]. RNA nucleotides provide three different interaction edges defined by the relative position of the polar atoms from the sugar and base rings: Watson-Crick, Hoogsteen and sugar edges [11]. In RNA structure, these chemical edges can interact in a canonical and non-canonical way via hydrogen bonds to generate several base pairing possibilities [6].

RNA folding is a relatively rapid process when compared with other biological time scales; small RNA molecules will fold in the range of pico to the milliseconds, whereas large RNAs may need minutes to hours to reach their functional three-dimensional structures [12]. Tertiary interactions and chain length will also modulate the accuracy and speed of RNA folding [13]. Taking into account the transcriptional speed of typical RNA polymerases (10–30 nt·s^−1^ for human RNA PolII, 50–100 nt·s^−1^ for bacterial polymerases and 200 nt·s^−1^ for viral polymerases), it is evident that the majority of nascent RNAs will undergo folding during transcription. First evidence of co-transcriptional folding was obtained by the study of ribozymes, showing that transcriptional pausing events also affect RNA folding [14,15,16]. Indeed the majority of RNA folding studies have been performed using* in vitro* models, and this fact has prevented a wider knowledge of the nucleic acid folding processes within cells [17,18].

## 2. Structural and Functional Families of RNA Helicases

Intramolecular interactions are the main driving forces that control the propensity of RNA to form secondary and tertiary structures. Some of these interactions are required for RNA maturation, localization or biological activity; however some of them could be also deleterious for its properties. The centrality of RNA molecules within cells has designed an ubiquitous and largely heterogeneous group of proteins, globally known as RNA chaperones or RNA helicases, that help RNA to reach and maintain its functional conformational state [3,19]. Some of these RNA helicases are chaperone-like proteins that prevent RNA to reach energy minima characterized by an incorrect conformational state during folding. Others are correctors of misfolded RNAs, able to resolve incorrect structural elements and to produce single stranded RNA. Interestingly, in higher eukaryotes, the RNA helicase family has also evolved to perform more specific tasks often comprising heterogeneous interaction partners that are tethered to specific RNA locations [20,21].

RNA helicases are RNA-binding proteins able to resolve secondary and tertiary RNA structures in an active manner, in some cases coupling this enzymatic activity to the hydrolysis of ATP [19,22,23]. This ubiquitous group of proteins is found in all the kingdoms of life, ranging from viruses to mammals, and it is closely related to DNA helicases. RNA helicases are included in five of the six nucleic acid helicase superfamilies; RNA helicases belonging to superfamilies SF3, SF4, and SF5 are oligomeric proteins (mostly hexamers), being typically encoded by genomes of viruses or bacteria [24]. Superfamilies SF1 and SF2 of RNA helicases are non-oligomeric proteins, containing a conserved bi-lobular core composed by two RecA-like domains as a central structure. Several sub-families of RNA helicases have been also defined on the basis of their sequence conservation and biological activity [23,25].

**Figure 1:**
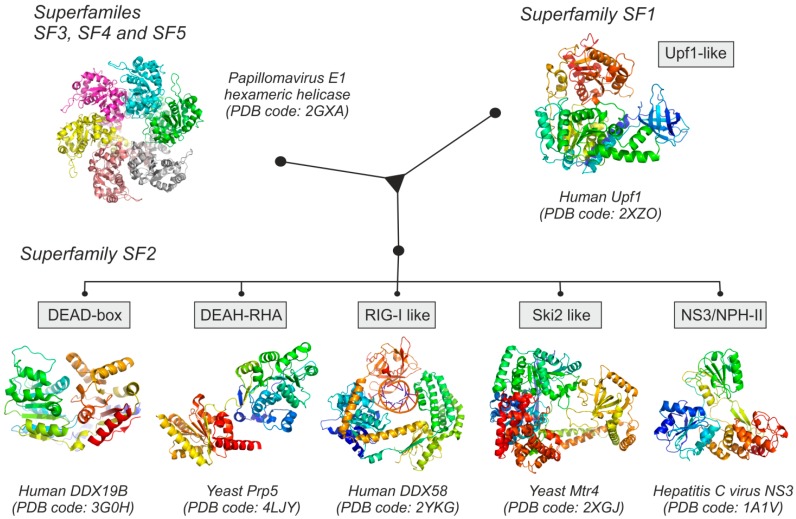
Structural families of RNA helicases, showing a representative Protein Data Bank (PDB) structure of each group. Superfamilies SF3, SF4 and SF5 are mainly hexameric helicases of viral origin. Superfamilies SF1 and SF2 are composed of monomeric proteins, found in all living organisms. RNA helicases belonging to SF2 are extremely diverse and can be sub-divided into five different classes, which only share the characteristic RNA-binding domain composed by RecA-like structural building blocks.

Human RNA helicases are included in five different sub-families: Upf1-like, DEAD-box, DEAH-RHA, RIG-I like and Ski2-like proteins (Figure 1) (See Table S1 for an updated structural information of human RNA helicases). The Upf1-like sub-family belongs to the SF1 superfamily, and includes a group of enzymes involved in RNA metabolism centered in processes like splicing or nonsense-mediated decay [26,27]. The SF2 superfamily includes five different sub-families of RNA helicases: DEAD-box helicases, DEAH-RHA helicases, RIG-I like proteins, Ski2-like proteins and the NS3/NPH-II subfamily, this last comprised only of proteins of viral origin. In addition to the presence of the characteristic helicase domains, SF1 and SF2 RNA helicases have other variable accessory domains located around their structural cores which frequently contain specific additional functionalities such as DNA-binding, protein-binding or oligomerization [28,29,30].

**Figure 2:**
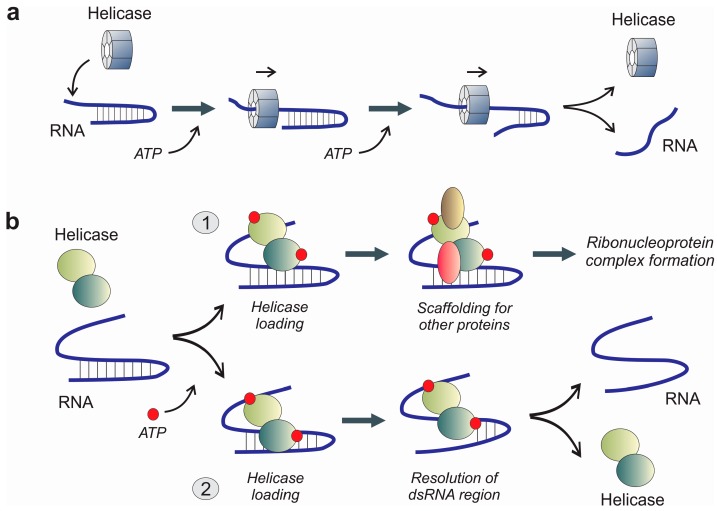
Schematic representation of the proposed reaction mechanisms of several groups of RNA helicases, showing the panoply of their biological activities. (**a**) Hexameric helicases belonging to SF3, SF4 and SF5 superfamilies are able to unwind and resolve RNA hybrids by coupling the ATP hydrolysis to an active mechanism that involves a displacement of the protein hexamer along the RNA strand; (**b**) Monomeric helicases are able to recognize specific RNA secondary structures; non-processive monomeric helicases will bind to dsRNA regions and use ATP to build protein-RNA complexes acting as scaffolds (1); on the other hand, processive monomeric RNA helicases (2) will resolve RNA secondary structures by a mechanism that typically involves ATP hydrolysis and a local action without displacement of the helicase along the RNA chain.

According to their catalytic properties and mechanism, two different types of RNA helicases can be distinguished (Figure 2). Some RNA helicases are able to catalyze a canonical unwinding of RNA duplexes, by a mechanism that involves the protein binding to a single-stranded region of the RNA and then translocating along the RNA duplex facilitated by ATP hydrolysis [19,22] (Figure 2a). This mechanism was observed in DNA and RNA hexameric helicases, and also in the Ski2-like, DEAH-RHA, Upf1-like and the viral NS3 families of monomeric proteins [31]. On the other hand, some monomeric helicases represented by the DEAD-box family of proteins constitute a completely different group in terms of catalytic properties, since they are able to resolve RNA duplexes by local strand separation (Figure 2b). Local helicase activity of DEAD-box proteins is based on the enzyme loading over the RNA duplex mediated by ATP binding. Upon enzyme loading, the RNA helicase is able to locally open the RNA duplex facilitating the formation of a single-stranded structure [32]. The proposed model for the catalytic activity of this group of RNA helicases suggests that the hydrolysis of ATP occurs before the strand separation. ATP hydrolysis is essential for the efficient release of the free enzyme from the RNA. The process is performed locally without any displacement of the enzyme along the RNA strands [33]. Moreover, some proteins harboring helicase domains are able to recognize specific patterns in RNA molecules, bind to them and act as a skeleton to build ribonucleoprotein complexes without a specific catalytic activity over the RNA secondary structures. However, the sequence and/or structure rules governing this recognition mechanism are far from completely understood. Among them, RIG-I and MDA5 cytoplasmic receptors of innate immunity are the most prominent examples.

## 3. DEAD-Box Helicases: RNA Folding Sentinels for a Myriad of Functions

### 3.1. Structural and Mechanistic Features of DEAD-Box Helicases

**Figure 3:**
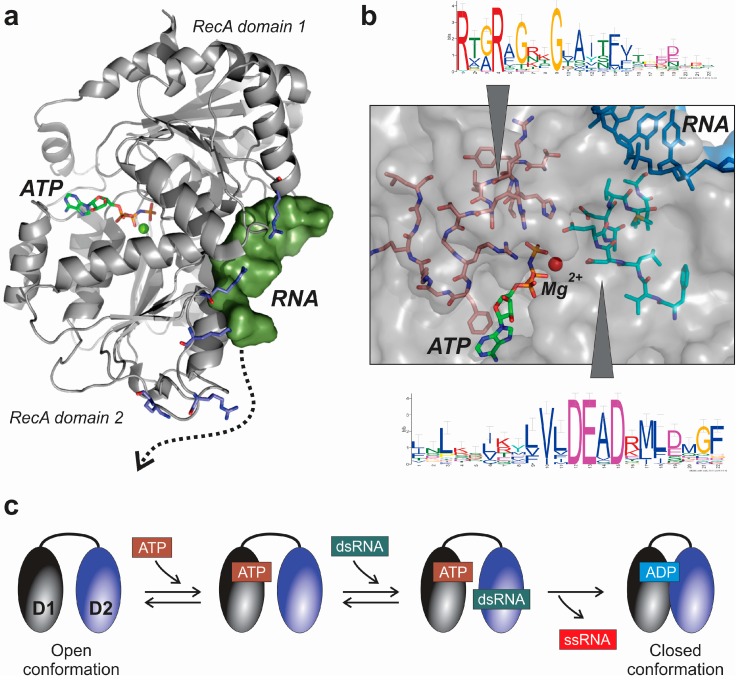
Structural features of the central core of DEAD-box RNA helicases exemplified by the structure of human DDX19B helicase (PDB code: 3G0H). (**a**) Structure of the human DDX19B protein, showing the ATP-binding cleft between both RecA-like domains, and the RNA binding site. The surface close to the RNA binding-site contains a string of positively-charged residues that could support the stabilization of protein-RNA binding along the helicase surface, following the path depicted as a dotted arrow; (**b**) Detailed representation of the location of the DEAD-box linear motif in the core of the DDX19B protein, close to the ATP-binding pocket. The core of the protein also contains an arginine-rich region which is involved in ATP-binding and stabilization of the movements of the RecA-like domains during RNA binding and unwinding; and (**c**) Molecular mechanism of dsRNA unwinding by yeast Mss116p helicase, as proposed from data obtained in X-ray crystallography experiments [40].

This class is the largest and most diverse group of RNA helicases. In terms of structural features they appear to be extremely redundant, since all of them share the presence of a well conserved helicase SF2 core that consists of two RecA-like domains separated by a flexible linker (Figure 3a). An ATP-binding site is located at the boundary between both RecA-like domains, which is generally available when the protein is bound to a double stranded RNA (dsRNA) [34]. Flexibility between both RecA domains is essential for the coupling of ATP hydrolysis to the unwinding activity of DEAD-box helicases [35]. The ATP-binding pocket, located within both domains, is only accessible to the cofactor in the so-called “open” conformation of the protein [34,36]. The RNA-binding motif is composed of a strongly positively charged surface cleft, which is able to bind a single stranded RNA with five or more oligonucleotides (Figure 3b). This RNA-binding region is complemented by several additional positively charged residues, which are able to support the stabilization of the helicase-RNA complex (Figure 3a) [34,37,38,39]. As described in several structural studies performed by co-crystallization of the DEAD-box helicase together with synthetic RNA oligonucleotides, the protein-RNA interfaces in those proteins is ensured by interactions with the phosphate backbone of the RNA molecule [40]. In consequence, and because no bases are involved in the interaction, a sequence-independent interaction is expected [41].

Recent data obtained from the co-crystal structure of yeast DEAD-box helicase Mss116p, revealed the molecular mechanism of dsRNA unwinding [40] depicted in Figure 3c. In Mss116p, each RecA-like domain interacts independent and sequentially with ATP and dsRNA. At the beginning of the catalytic cycle with the helicase in the open conformation, ATP binds to the *N*-terminal domain 1, and duplex RNA to *C*-terminal domain 2 (Figure 3c). The protein-ATP-RNA hybrid will transit to the closed conformation by ATP hydrolysis and RNA displacement facilitated by the domain closure. Since the helicase in closed conformation is not compatible with the binding to a dsRNA, the unwinding of the RNA is most likely to occur during this transition from the open to the closed protein forms [37,42]. Interestingly, ATP hydrolysis appeared to be required for an efficient release of the protein from the RNA, and thus a productive enzyme turnover, and not for the dsRNA unwinding [43]. However, some very recent evidence obtained from yeast Hera and YxiN RNA helicases showed that there might be some exceptions to the general catalytic domain already described [37]. In fact, Samatanga and Klostermeier determined that both RecA-like domains from Hera and YxiN helicases are able to bind dsRNA [37], in contrast to the described mechanism observed in Mss116p protein [40]. In consequence, some DEAD-box helicases showed a cooperative mechanism involving both RecA domains, where the binding to dsRNA is not an exclusive function of the *C*-terminal RecA-like domain [37].

Recently, Pan and coworkers [44] demonstrated the relationships between the RNA stability and the unfolding efficiency using the CYT-19 protein, a mitochondrial DEAD-box RNA helicase from *Neurospora crassa*, as a model. By using single molecule fluorescence, the authors were able to show that the ability to sense RNA stability probably biases DEAD-box helicases to act preferentially over less stable misfolded RNA structures, promoting folding and diminishing useless interactions with folded RNAs.

**Figure 4:**
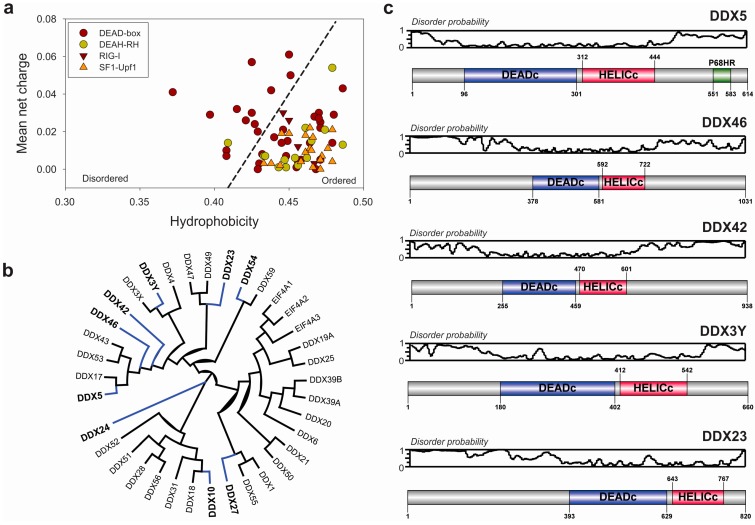
Evidence of the presence of disordered segments in human DEAD-box RNA helicases. (**a**) Uversky plot: hydrophobicity *vs.* mean net charge plot of human RNA helicases (from [11]). Members of each sub-family of proteins are depicted by a different symbol as indicated in the legend panel. Intrinsically disordered proteins are recognizable by a combination of low hydrophobicity and higher mean net charge. The order-disorder boundary line is defined by the linear function < charge ≥ 2.743 < hydrophobicity > −1.109 [55]; (**b**) Circular chladogram obtained by multiple sequence alignment of human DEAD-box helicases by Multalin software [56] and represented in Dendroscope package [57]. Proteins labeled in black and connected by blue tree branches contain more than 45% of residues located within intrinsically disordered segments, as determined by Foldindex algorithm [58]; (**c**) Detailed analysis of the probability of the presence of disordered segments in selected human DEAD-box helicases. Probability of disorder of each amino acid along the protein sequence was determined by the PrDOS algorithm [59], and represented on the top of a linear domain map for the analyzed DEAD-box RNA helicases. Depicted data clearly shows the presence of a structured core helicase domain flanked by disordered and flexible segments that could be putatively involved in protein-protein interactions.

DEAD-box helicases have other variable accessory segments located flanking the helicase structural and functional core which frequently confer additional specific functions such as DNA-binding, protein-binding or oligomerization [28,29,30]. Analysis of human RNA helicase sequences showed that the majority of these enzymes belong to the class of globular proteins [45]. Interestingly a particular group of RNA helicases with a high content in structurally disordered segments has been found within DEAD-box helicases (Figure 4a).

The majority of RNA helicases analyzed* in vitro* do not display a clear sequence or structural preferences for their substrates. Despite this lack of substrate specificity, the majority of RNA helicases works on unique cellular process [46,47,48]. Disordered segments might be likely involved in protein-protein interactions, modulating the RNA helicase specificity for its substrate by recruitment of different partners. The presence of flexible long stretches would be also related to the interaction promiscuity, as previously described [49,50]. Considering the group of human DEAD-box helicases, the analysis of the probability of disorder by individual residues showed the presence of nine members of the group containing more than 45% of their amino acids within predicted disordered segments (Figure 4b). This fact has been recently demonstrated in a different family of proteins; Hef an archaeal DNA helicase involved in DNA repair contains intrinsically disordered segments that interact with several different proteins that work together in the DNA repair mechanism [51]. The presence of interacting disordered segments in nucleic-acid binding proteins could be extensive in the RNA-helicase field [39]. Structural studies of these auxiliary domains for DEAD-box helicases have been prevented by their intrinsic flexibility, with a few exceptions in bacterial helicases where some of those accessory domains were related to dimerization and protein interactions [52,53,54].

### 3.2. Extended Functional Paradigm of DEAD-Box Helicases

Besides classical RNA unwinding activity, DEAD-box RNA helicases have shown a high degree of sophistication to adapt themselves to other biological functions, taking advantage of their conserved characteristics. Putnam and Jankowsky, in a recent review, discussed the idea that DEAD-box RNA helicases might be integrators of several biological processes since they can act within the interfaces of RNA and protein metabolism [60]. Among the diverse functions that can be associated with DEAD-box RNA helicases, we can cite, among others, their role as anchors for the assembly of macromolecular ribonucleoprotein complexes, displace proteins from RNA and act as molecular sensors for primary metabolites [61,62,63].

DEAD-box helicases can disrupt RNA-RNA and protein-RNA complexes. Disruption of a molecular complex by a DEAD-box helicase is dependent on the dissociation constant of the already formed complex, being a competitive process controlled by equilibrium dynamics [44]. This mechanism was previously demonstrated and characterized in DExH/D helicases [62]. Probably one of the best characterized cases of molecular displacement mediated by DEAD-box helicases is the ribosome biogenesis. In this process, the DDX51 helicase binds to pre-60S subunit complexes and promotes displacement of U8 snoRNA from pre-rRNA, which is necessary for the removal of the 3'-external transcribed spacer from 28S rRNA and productive downstream processing [64]. However, the mechanistic analysis of protein displacement in complexes by DEAD-box helicases has not been reported until now, probably because of the transient nature of some complexes where those proteins are involved.

Another complementary function of the DEAD-box helicases is to serve as anchors for the assembly of macromolecular complexes. In this context, the function of the helicase would be to act as a bridge between the RNA and the protein members of the complex. This phenomenon has been generally defined as “clamping” [60] and was characterized for the first time in the DEAD-box protein eIF4A-III, which acts as a platform for the assembly of the multicomponent exon-junction complex [61]. The crystal structure of the exon-junction complex to 2.3 Å resolution was determined, showing the molecular role of the eIF4A-III helicase as a platform for the assembly of the complex around a single-stranded RNA, and also as a homeostatic factor that avoids the formation of structured RNA segments [65]. Recent evidence also showed the ability of the yeast DEAD-box helicases Mss116p and Ded1p to form long-lived stable complexes over RNA molecules, in an ATP-dependent fashion [66].

Interestingly, a restricted group of DEAD-box helicases appeared to be sensitive to AMP levels, which are also potent inhibitors of their unwinding activity under normal conditions. The yeast DEAD-box helicases Sub2p and Dbp5p are not inhibited by AMP. This family of DEAD-box helicases can potentially act as internal biosensors with the capacity to directly link changes in AMP concentrations to RNA metabolism [67]. The roles of these putative AMP sensors still need to be investigated in more detail.

Additional roles of DEAD-box helicases, yet to be explored, are the characterization of their functions in relationship with the non-coding transcriptome. Besides the small non-coding RNAs, it is well known that the eukaryotic genomes contain a variable number of transcriptional units devoted to the production of large non-coding RNAs (lncRNAs). This class of RNAs are >200 bp in length, lacking significant protein coding capacity. The biological roles of lncRNAs are diverse, ranging from the transcriptional control of gene expression at the chromatin level to the interactions with other regulatory RNAs as miRNAs [68,69]. In some well documented cases, lncRNAs act as scaffold of macromolecular complexes, or can guide proteins to perform their catalytic or regulatory functions in the right cellular locations [70,71]. Since lncRNAs functions are highly dependent on their structure, it is very tempting to speculate about the possible role of RNA helicases on the global non-coding RNA homeostasis. However, our knowledge of the role of RNA helicases on lncRNAs function is still very limited, and only supported by a few experimental studies. Among them, the role of RNA helicases in the functional dynamics of splicing by their interaction with spliceosomal ribonucleoproteins (RNPs) is well established, and reviewed elsewhere [72]. Probably the most striking results supporting the functional relationships between RNA helicases and non-coding RNAs came from the virology field, and are currently being extended to other areas. In fact, DDX3 and DDX5 helicases are crucial players in the replicative cycle of some viruses, since they are able to interact with non-coding RNA regions of the viral genome. In Japanese encephalitis virus (JEV), DDX3 helicase from host cells interacts with the 5' and 3' non-coding ends of the viral RNA genome enhancing viral RNA translation, which might affect viral RNA replication at the late stage of virus infection [73]. Moreover, DEAD box helicase DDX5 is also one of the better known examples of helicases involved in lncRNA metabolism. Recent evidence suggested that DDX5 is an important regulator of the expression of a subset of miRNAs in breast cancer cells, including miR-21, an oncogenic and tumor-promoting miRNA [74]. Moreover, DDX5 is also frequently associated to steroid receptor RNA activator (SRA), a lincRNA, to form a complex with CTCF that is essential for the function of the chromatin insulator [75]. Chromatin insulators are DNA-binding complexes that influence eukaryotic gene expression by organizing the chromatin into transcriptional territories [76]. Interaction between DDX5 helicase and its cognate lncRNA SRA has been also related to the activation of the Notch signaling pathway [77].

## 4. RNA Sensors: The RIG-I Family of Helicases

The RIG-I family of helicases is composed of multidomain proteins that belong to the innate immune system, a well conserved first line of defense against pathogens. The mission of the innate immune system is to generically detect pathogen-associated molecular patterns (PAMPs), which include RNA molecules harboring stable secondary structures as double-stranded regions, but also other molecules from the external surface of the invading agents. Defense lines integrated within the innate immunity comprise the presence of extracellular and intracellular receptors. The RIG-I-like family of proteins (RLRs: RIG-I-like receptors) are part of the cytoplasmic receptors for PAMPs, interacting with nucleic acid molecules from infecting viruses [78]. RLRs act as signal transducers after the interaction with their cognate RNA targets. The transmission of their defense signal throughout the cell is mediated by the recruitment of several other players that will lead to an overall interferon-mediated response and a triggering of the mitochondrial antiviral signaling cascade (MAVS) [79,80].

The RIG-I family of cytoplasmic receptors is composed of the retinoic-acid inducible gene (RIG-I, DDX58), the melanoma-differentiation associated (MDA5) and the LGP2 genes. Besides these genes, the RIG-I family of helicases also includes DICER, a hybrid helicase-nuclease involved in the biosynthesis of miRNAs and the RNA interference process [81,82]. The RIG-I family of proteins can be considered as product of the evolution that selected some of the functions of the helicases in detriment to others [83]. Taking into account the deep conservation of these cellular players of the innate immune system, we can postulate that they may have evolved together with other RNA-binding proteins, selecting the helicase domain to bind dsRNA [84,85]. In consequence these receptors cannot be strictly considered as proper helicases; however they harbor a characteristic bi-lobular helicase domain with a conserved ATP-binding site.

The RIG-I protein, the main representative of this protein family, was initially identified as a coding transcript associated with retinoic acid-induced differentiation of acute promyelocytic leukemia cells, being included in a wider family of interferon-stimulated genes [86]. RIG-I is a large multidomain protein, conserved in many eukaryotic cells ranging from protozoa to humans. The *N*-terminal region is composed of two caspase-recruitment domains (CARDs), followed by the typical helicase core region formed by two RecA-like domains. The *C*-terminal region of the protein contains a regulatory domain, characteristic of this family, which harbors a structural Zn atom [78,79]. RIG-I is activated by a wide family of RNAs produced by viral metabolism [87,88]. The scientific community is not in agreement concerning the minimal requirements that those RIG-I-activating RNAs must harbor, but it is believed that RIG-I can be activated by RNA molecules containing a 5'-triphosphate nucleotide together with a blunt-ended base paired region at the 5'-end of the RNA molecule [89,90]. Structural evidence suggests that a lysine-rich cleft within the RIG-I carboxy-terminal domain (CTD) domain is responsible for the sequestration of the terminal 5'-triphosphate by a polar interaction [91]. Recently, Goubau and coworkers [92] showed that RIG-I also mediates antiviral responses to RNAs bearing terminal 5'-diphosphates (5'-pp).

**Figure 5:**
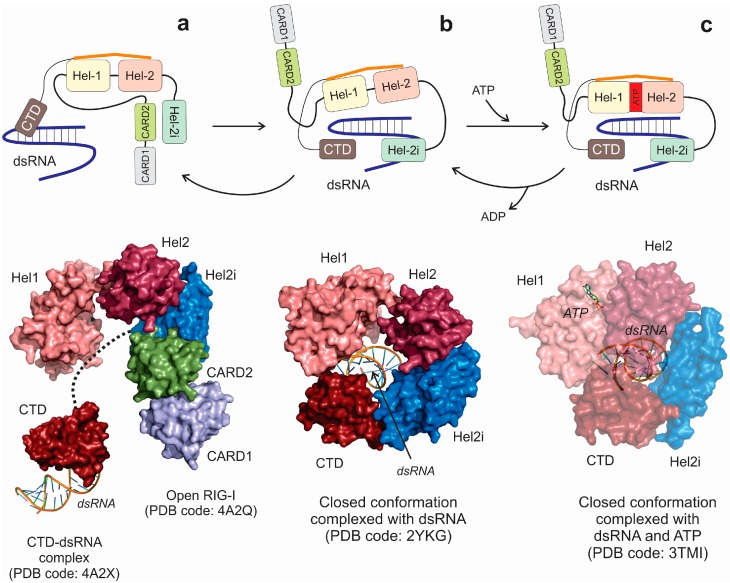
Structural interpretation of the mechanism of RIG-I activation by dsRNA. (**a**) In an initial step, the protein is in an open conformation and the exogenous viral dsRNA molecule is recognized by the CTD domain. The binding of a dsRNA to the CTD domain will induce a dramatic change in protein conformation, which will produce the establishment of closer contacts between the dsRNA and the Hel2i domain and the displacement of the CARD domains that will be available for further downstream interactions (**b**). Interestingly, in the absence of ATP this complex appears to be very unstable and has the tendency to revert to the depicted open conformation; (**c**) ATP binding to the inner pocket between Hel1 and Hel2 domains will stabilize the closed conformation of the protein complex, which is believed to be the active form of the receptor that will act as a downstream signal transducer.

Structural studies of RIG-I protein have been delayed for a long time since the protein seemed to be resistant to crystallization. Finally, in 2011 at least three independent groups reported different RIG-I crystal structures in complex with synthetic RNAs [79,84,87]. The most detailed work was published by Kowalinski and coworkers [79] who determined the tridimensional structure of duck RIG-I in several conditions, including an open conformation, a dsRNA complex and a closed ATP-dsRNA complex. As a whole, the structural studies on RIG-I complexes have been essential for the dissection of the molecular mechanism of receptor activation, partially depicted in the selected snapshots of Figure 5. In the absence of exogenous viral infections, the RIG-I receptor is in its auto-inhibited state, where the *N*-terminal CARD domains are interacting with the Hel2i domain, blocking the RNA-binding pocket formed between the Hel1 and Hel2 domains (Figure 5, panel a). On the other hand, under these conditions the CTD domain is connected with a flexible linker to the core of the receptor, becoming available for the scanning of potential viral RNA targets [85]. In the event of the presence of a proper viral RNA target within the cell, the RNA is detected and blocked by the CTD domain, and the formation of a closed protein-RNA complex using the RNA-binding pocket constituted by Hel1, Hel2 and Hel2i domains follows. This movement of protein domains will displace the *N*-terminal CARD segments, becoming free for further protein interactions (Figure 5, panel b). Interestingly, the stability of this initial protein-RNA complex is low in the absence of ATP, being prone to return to the auto-inhibited state [84,85]. The signaling-competent state of the complex is ensured by the binding of an ATP molecule between Hel1 and Hel2 domains, which will lead to a tighter interaction between Hel domains around the target dsRNA molecule [79,80,84] (Figure 5, panel c).

Moreover the innate immune system is complemented by the presence of other RIG-I partners. Among them, MDA5 is the most prominent one, acting as a helper of RIG-I in the detection of viral RNAs [88]. In humans, MDA5 protein is also a receptor for sensing dsRNAs, which shares sequence homology and structure with RIG-I (Figure S1). The roles of MDA5 seem to be non-redundant and complementary to RIG-I, acting by the same molecular mechanism. As evidenced by early functional studies, MDA5 was able to cooperatively assemble into a filamentous oligomer composed of a repeating segmental arrangement of MDA5 dimers along the length of the target dsRNA [93]. X-ray crystallography methods recently solved the molecular structure of MDA5 in complex with RNA [94]. MDA5 has a modular architecture similar to RIG-I, however the *N*-terminal CARD domains are separated from the helicase core by a stretch of 40 amino acids, which is absent in RIG-I (Figure S1). MDA5 showed an open ring shape when complexed with synthetic dsRNAs, which is compatible with the protein binding to the stretch of the dsRNA fiber observed in the initial experiments by Peisley and coworkers [93]. In fact, the decoration of viral dsRNA fibers by MDA5 is ensured by the dimerization capabilities demonstrated by the CARD domains, and coupled with ATP hydrolysis [84,94]. The MDA5 oligomerization induced by the presence of viral RNAs has been shown to be essential for MAVS activation [94]. However the molecular mechanism of dynamic and synergic interactions between RIG-I and MDA5 are still not completely understood.

## 5. RNA Helicases Involved in Viral Infections

Viral replication within host cells is an intrinsically demanding process, which requires the hijacking of the cellular machinery by the virus. The spatial-temporal accumulation of high levels of metabolic stress induced by the virus, obligate the host cell to increase its normal pace in processes such as transcription and translation. In general we can affirm that viral infections are highly RNA-dependent processes, due to the central position of RNA within cellular metabolism. The viruses have evolved together to their host cells, developing mechanisms to control and guarantee the success of their replicative cycles. Moreover, for many important functions, viruses encode proteins closely related to host proteins or have the ability to directly steal cellular factors performing the same functions [95,96,97].

The already discussed RNA-dependence of the viral infection has prompted the necessity of efficient RNA-helicases to ensure proper viral replication. Despite other considerations, our structural knowledge of viral helicases has been guided by the relative importance of selected viruses in human health, since viral helicases are potential targets for the rational design of antiviral drugs [98,99,100]. Probably, the most widely characterized family of viral RNA helicases is composed of the NS3 proteins (non-structural protein number 3) from the *Flaviviridae* family. Flaviviruses are single-stranded RNA viruses of positive polarity that can be etiological agents of major diseases such as Hepatitis C, yellow fever, Japanese encephalitis and dengue. In these viruses, NS3 protein is a bi-functional protease-helicase with a central role in the replicative cycle of the virus [101]. Structural information based on X-ray crystallography experiments is available for several NS3 helicases, as depicted in Table 1.

**Table 1:** Representative structures of NS3 helicases from elements belonging to the *Flaviviridae* family of viruses.

Virus	Structure	PDB Code
Dengue virus	Full length helicase-protease apo-enzyme	2JLQ
	Helicase core complex with AMPPNP	2JLR
	Helicase core complex with ADP	2JLS
	Helicase core complex with ssRNA	2JLU
	Helicase core complex with ssRNA and ADP	2JLZ
Yellow fever virus	Helicase core	1YKS
	Helicase core complex with ADP	1YMF
Murray valley encephalitis virus	Helicase core	2WV9
Kunjin virus	Helicase core	2QEQ
Japanese encephalitis virus	Helicase core	2Z83
Hepatitis C virus *	Full length helicase-protease apo-enzyme	3O8C
	Full length helicase-protease complex with ssRNA	3O8R
	Full length helicase-protease complex with a protease inhibitor	4A92
	Helicase core complex with ssRNA	3KQU

* Due to the clinical significance of HCV infections, the number of PDB entries is much larger, including complexes with specific inhibitors for the protease and helicase domains.

**Figure 6:**
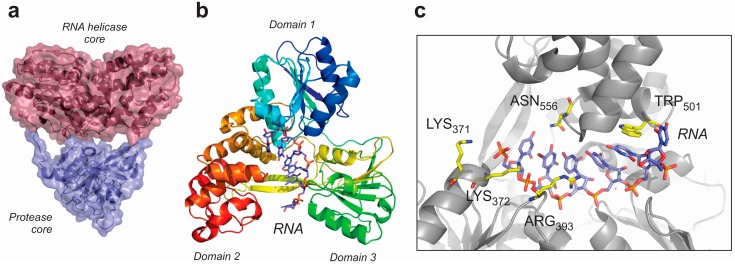
Structural features of viral RNA helicases from the *Flaviviridae* family, exemplified by the structure of the NS3 bifunctional protease-RNA helicase from human hepatitis C virus (HCV). (**a**) Overall structure of the bifunctional NS3 protein, showing the location of the protease and helicase cores (PDB code: 4A92); (**b**) Structure of the RNA helicase core from the HCV NS3 protein in complex with a synthetic RNA oligonucleotide (PDB code 3KQK); and (**c**) Detail of the residues involved in the interaction with RNA in the HCV NS3 helicase core. Note the presence of positively charged residues (Lys_371_, Lys_372_ and Arg_393_) interacting with the phosphate backbone of the RNA molecule. The RNA binding pocket is completed by amino acids interacting with the RNA bases (Asn_556_ and Trp_501_), which will ensure a relative substrate specificity [104].

NS3 proteins are composed of two cores harboring two different activities: RNA helicase and protease. The helicase core contains three domains (Figure 6a,b). Interestingly, structural data showed that two of these domains (Domains 1 and 2, Figure 6b) are structurally similar to those characterized in DEAD-box helicases [102,103]. X-ray structures of several NS3 helicases in complex with synthetic oligonucleotides showed that the RNA-binding cleft is located in the interface between domains 1 and 2 [104,105,106]. In HCV helicase, it has been possible to characterize the whole RNA unwinding mechanism by the determination of the structures of the apo-enzyme and several complexes with RNA and non-hydrolysable analogues of ATP. Results indicated that NS3 helicase from HCV unwinds RNA molecules by a translocation mechanism which consumes one molecule of ATP per each resolved base pair [104]. The NS3 helicase translocates along the RNA by a “spring-loading” mechanism starting from the 5'-end of the RNA, using a central Tryptophan residue (Trp_501_), which interacts with the RNA bases (Figure 6c). Interestingly, the RNA-binding cleft in NS3 helicases is comparatively narrower than the same structure in the DEAD-box family, and the protein-RNA interaction is ensured by contacts either with the phosphate-sugar skeleton or the bases simultaneously [102,107,108]. Another differential characteristic of the NS3 helicases is the presence of an additional domain in the helicase core, which confers to the enzyme a tri-lobular shape. The function of this third domain is still not completely understood, but molecular dynamics simulations pointed out the possibility that it can be involved in the allosteric coupling between the helicase and protease activities. Moreover, the disruption of the interface between protease and helicase cores did not significantly alter the RNA unwinding activity of the protein. However, the full-length polypeptide was more efficient, suggesting a probably direct role of the interface between structural cores in both of the catalytic activities of the NS3 protein [109].

Due to the relevant role of NS3 protein in the Flavivirus infection cycle, the use of structural biology techniques to design new potential inhibitors of this protein has been essential during the last decade to put onto the market new drugs with antiviral activity [110]. Classical drugs such as Telaprevir or Boceprevir are competitive inhibitors of the protease domain of NS3 used for the treatment of hepatitis C, and have been designed by taking advantage of structural information and rational drug design methods [111,112,113]. However, the outbreak of resistances based on specific mutations of the protease domain [111,114] have facilitated the development of new inhibitors targeting the helicase domain of the NS3 protein [101,115]. Organic compounds [116,117], antibodies [118] and RNA aptamers [119,120] used as inhibitors of the helicase domain have already been described. A new wave of future NS3 inhibitory drugs would be a reality in a next future designed by simultaneously targeting both of the enzymatic activities [98].

## 6. Conclusions and Further Perspectives

RNA folding is a dynamic, energy demanding and complex process where RNA helicases are main players. In the context of the pervasive transcription of the genome, the RNA helicases have gained more relevance as essential players in cell homeostasis. Interestingly, their functions are far from being restricted to simple co-adjuvants of the energy-driven RNA folding process, since they are an extremely diverse family of proteins. Many RNA helicases have ubiquitous location and function within the cell, exemplified by the paradigmatic case of RIG-I helicase as a master trigger of the innate immune response [121]. In particular conditions, RNA helicases could even be used by the cell to sense small molecules such as AMP [67], and some of them might have an essential role in the definition of gene expression regions of chromatin [77]. Furthermore, the characteristic RNA-binding properties of this family of proteins make them suitable to complex functions, participating in tangled regulatory mechanisms. To contribute to their intricacy, some of the RNA helicases contain long disordered stretches, which might be involved in protein-protein interactions and probably could be important factors modulating their substrate specificity and functional activity.

Structural biology has been essential to dissect many of the RNA helicase functions, mainly focused in the characterization of their catalytic activities. However, the intrinsic nature of these proteins has prevented the structural characterization of some of their biological partners. A multidisciplinary approach will be required to characterize more helicase-containing complexes, including those built over large RNA-molecules, instead of synthetic oligonucleotides. For the characterization of those complexes a combination of X-ray crystallography and high-resolution cryo-electron microscopy together with RNA-mapping techniques should be the most adequate strategy. Recently, this approach has been successfully applied for the characterization of large RNA-protein complexes, as described in the characterization of the *E. coli* Cascade surveillance complex [122,123].

However, many questions related to this cohort of proteins remain to be answered, including the determinants of specificity (if they exist) for each particular RNA helicase and the additional functions of these proteins rather than those related to RNA folding. Also a matter of further studies will be the presence of putative groups of functionally specialized RNA helicases, devoted to the control of the homeostasis of selected groups of RNAs such as non-coding RNAs. High-throughput techniques will also be essential for the characterization of RNA helicase function in several contexts, especially for the determination of their roles in the pervasive transcription and the homeostasis of non-coding RNAs. Recently a new family of methods that combine high-throughput sequencing with RNA structure probing has been developed [124,125]. These methods allow the determination of hundreds of RNA structures* in vivo* in a genome-wide fashion, and will contribute to the overall understanding of the physiological functions of RNA structures. RNA probing combined with next-generation sequencing (NGS) comes in several flavors, namely methods such as SHAPE-seq [126], Structure-Seq [124], Mod-seq [127], Dash-seq [128], or SPOT-seq-RNA [129]. The subjacent idea of* in vivo* RNA structure determination is based on the chemical or enzymatic probing of secondary RNA structures followed by a sequencing step. This powerful family of methods is however limited by the intrinsic characteristics of the RNA-seq protocols, and also by the structural diversity of RNA molecules. Methods such as Structure-seq [124] and Mod-seq [127] are based on the chemical probing of RNA secondary structures by the use of dimethyl-sulfate (DMS). DMS is a small molecular weight reagent, compatible with living cells, and able to methylate specific nitrogen atoms in structurally exposed A, C and G bases within RNA. Methylated A and C bases inhibit the reverse transcription step prior to sequencing, since the methylation modifies the Watson-Crick interaction plane of the bases, and this inhibition can be consequently mapped by RNA sequencing [127]. Combining this information with the computational predictions of secondary structure elements, it is possible to obtain a global map of RNA structures within a cell [124,125]. Other methods such as SHAPE-seq combine selective 2'-hydroxyl acylation analyzed by primer extension with multiplexed paired-end deep sequencing to determine hundreds of RNA structures simultaneously [126,130]. These NGS-based structural protocols, combined with classical molecular and cellular biology techniques could be applied to determine the role of a particular RNA helicase in RNA homeostasis.

Thus, this family of widespread proteins is composed of essential players within the core of the cellular transfer of information and constitutes a very exciting scientific area, which has been empowered by the rising importance of the non-coding genomic output. The already described roles of RNA helicases in some human diseases will certainly open new research areas with the aim of characterizing the functional factors that regulate RNA homeostasis within the cell and their relationships with the onset and progression of RNA-dependent diseases.

## Supplementary Materials

Supplementary materials can be found at http://www.mdpi.com/1422-0067/16/02/2269/s1.
